# Near infrared light decreases synaptic vulnerability to amyloid beta oligomers

**DOI:** 10.1038/s41598-017-15357-x

**Published:** 2017-11-08

**Authors:** Michele M. Comerota, Balaji Krishnan, Giulio Taglialatela

**Affiliations:** Mitchell Center for Neurodegenerative Diseases, Department of Neurology, University of Texas Medical Branch, Galveston, TX USA

## Abstract

Synaptic dysfunction due to the disrupting binding of amyloid beta (Aβ) and tau oligomers is one of the earliest impairments in Alzheimer’s Disease (AD), driving initial cognitive deficits and clinical manifestation. Consequently, there is ample consensus that preventing early synaptic dysfunction would be an effective therapeutic strategy for AD. With this goal in mind, we investigated the effect of a treatment of mice with near infrared (NIR) light on synaptic vulnerability to Aβ oligomers. We found that Aβ oligomer binding to CNS synaptosomes isolated from wild type (wt) mice treated with NIR light was significantly reduced and the resulting suppression of long term potentiation (LTP) by Aβ oligomers was prevented. Similarly, APP transgenic mice treated with NIR showed a significant reduction of endogenous Aβ at CNS synapses. We further found that these phenomena were accompanied by increased synaptic mitochondrial membrane potential in both wt and Tg2576 mice. This study provides evidence that NIR light can effectively reduce synaptic vulnerability to damaging Aβ oligomers, thus furthering NIR light therapy as a viable treatment for AD.

## Introduction

Alzheimer’s disease (AD) is the most common and severe age-associated neurodegenerative disorder for which there is currently no effective therapeutic intervention. One of the key events contributing to the development of the cognitive decline that marks the clinical profile of AD is the selective targeting and disruption of the synapses by small, soluble amyloid beta (Aβ) oligomers, the most toxic form of the Aβ protein. Aβ oligomers binding to synapses have been shown to induce many morphological and physiological changes that collectively lead to the loss of cognitive integrity including retraction of synaptic spines^1,2^, decreased pCREB and increased calcineurin protein levels^3^ and reduced long term potentiation (LTP) initiation^4–6^. Furthermore, we recently reported a group of individuals referred to as **N**on- **D**emented with **A**lzheimer’s **N**europathology (NDAN) that provides evidence supporting the correlation between the presence of Aβ oligomers at the synapses and the retention of cognitive function. NDAN individuals maintain their cognitive integrity despite the presence of Aβ plaques and neurofibrillary tangles at an extent comparable to demented AD patients. By comparing these individuals to AD patients, one can infer properties that may protect these individuals from the cognitive dysfunction normally associated with AD pathology. We showed that while NDAN individuals displayed similar levels of soluble Aβ oligomers throughout their CNS, contrary to demented AD patients, they had synapses that were devoid of Aβ oligomers^7^. This suggests the possibility that synapses of NDAN subjects are resistant to Aβ oligomers and illustrates that absence of Aβ at synapse is a key event associated with preservation of cognitive integrity. Taken together this evidence suggests that eliciting a protective mechanism resulting in synaptic resilience to binding of Aβ oligomers similar to NDAN individuals would be the most effective protection against Aβ oligomer driven toxic synaptic dysfunction. With this goal in mind, in the present work we investigated the effect of a treatment with near infrared light (NIR, 600 to 1000 nm wavelength) in increasing synaptic resilience to Aβ oligomers. NIR light treatment administered in a noninvasive transcranial application has been suggested to photostimulate the mitochondrial chromophore, cytochrome c oxidase, resulting in increased ATP formation^8–11^. Mitochondrial dysfunction is a pathological event occurring in early stages of AD. Much evidence suggests dysfunctional mitochondria events such as increased reactive oxygen species (ROS) production^12,13^, cytochrome c deficiencies^14,15^, and mutated mitochondrial DNA^16,17^ contributes to the exacerbation of AD pathology^18,19^. Notably, studies have described the intimate relationship between reduced Aβ oligomer binding at synapses and increased mitochondrial function^20^. Thus, it is not unreasonable to hypothesize that NIR light treatment could effectively promote synaptic resilience to Aβ oligomer binding. To test this hypothesis, we investigated the ability of NIR light to reduce synaptic susceptibility to Aβ oligomer binding and, as a result, increase synapse function. We utilized wild type (wt) mice to determine the impact of NIR light treatment on the binding of Aβ oligomers to isolated synaptosomes and long term potentiation (LTP) in the hippocampus of these NIR light-treated wt mice in the presence and absence of Aβ oligomers. To determine light-driven changes in the synaptic presence of endogenous Aβ oligomers, we also investigated the Aβ_1–42_ load at isolated synapses of NIR light-treated Tg2576 mice that overexpress human amyloid precursor protein (APP) and progressively accumulate Aβ in their CNS^21–24^. We further investigated synaptic mitochondria in wt and Tg2576 mice after NIR light treatment. We found that NIR light treatment reduced *ex vivo* Aβ oligomer synaptic binding in wild type mice that was paralleled by a retention of long term potentiation induction. We further found that after NIR light treatment, the levels of Aβ_1–42_ at the synapses was significantly reduced in 6-month-old Tg2576 mice. These changes were in conjunction with a retention and increase in the synaptic mitochondria health after NIR light treatment in both wt and Tg2576 mouse models.

## Results

### Decreased susceptibility of synapses to toxic Aβ oligomers binding in wild type mice treated with NIR light

**Figure 1:**
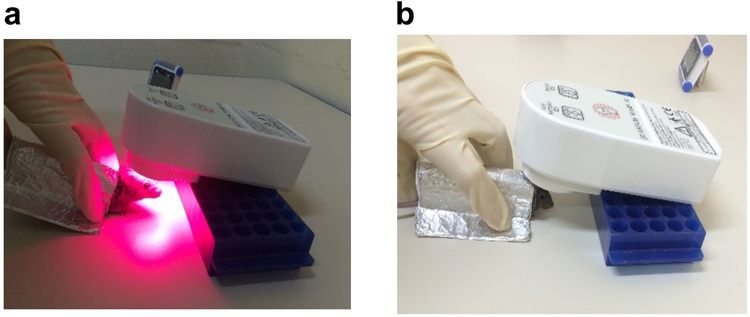
Near infrared light treatment procedure. The mice were treated with NIR light from a 670 nm light-emitting diode (LED) device. (**a**) The device was held approximately 1 cm above the head of the mouse while aluminum foil was held over the body to minimize NIR light exposure to the periphery. The mice received 1–90 second treatment per day for 5 day in a week over 4 consecutive weeks. (**b**) The control sham treatment group was held under the NIR light device in the same manner as the NIR light treated group with the device remaining off.

**Figure 2:**
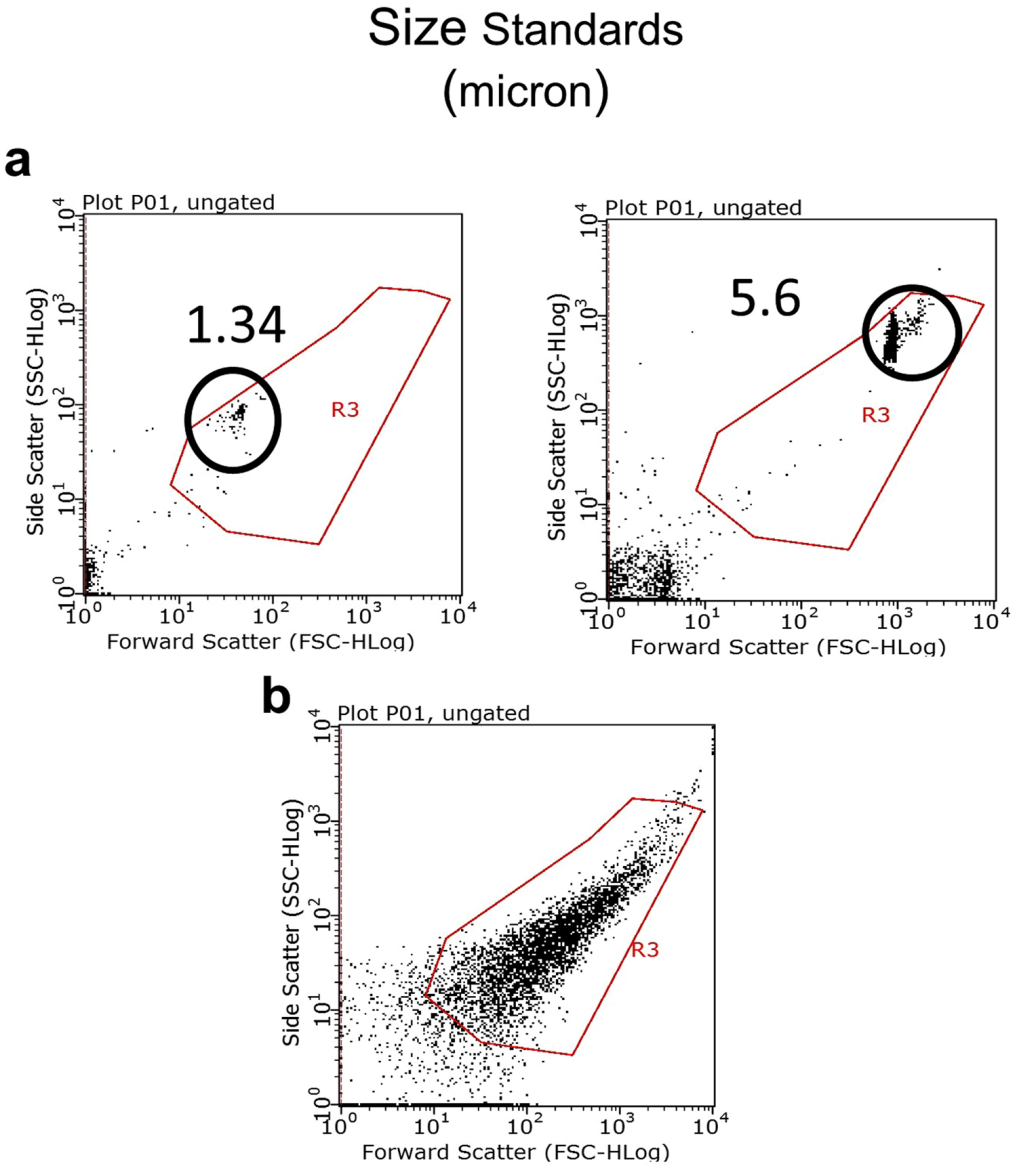
Flow cytometry gating parameters. (**a**) Size beads (circled) ranging from 1–5.6 μm, the average size range of synaptosomes, were used to determine the gate (red box). (**b**) The size gate was applied to the particle distribution of mouse synaptosome preparation to exclude non-synaptosomal particles.

**Figure 3:**
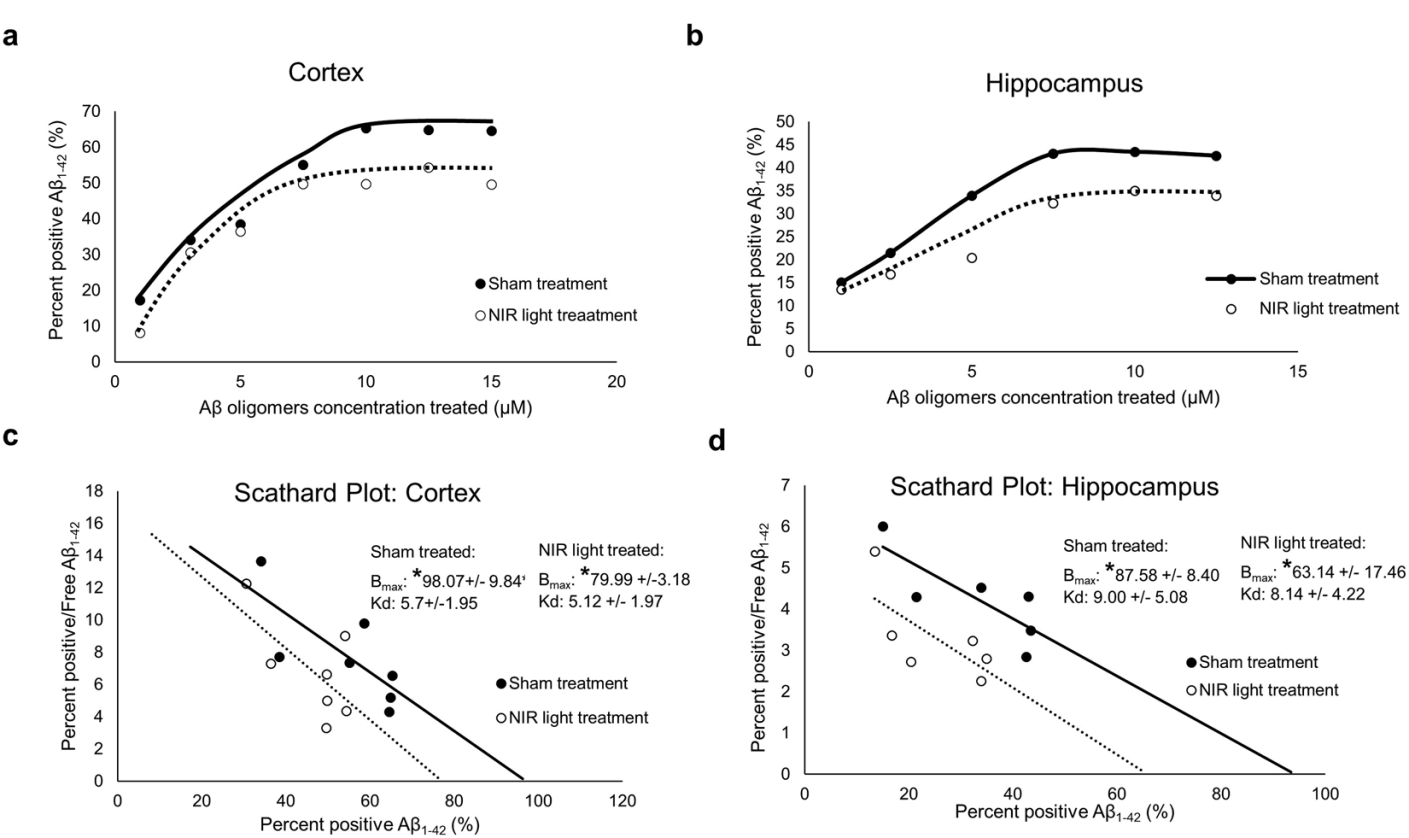
Flow cytometry analysis of NIR light treatment Aβ oligomer binding curve. Groups of pooled synaptosomes from (**a**) cortex and (**b**) hippocampus of WT mice receiving NIR light treatment (black square) and sham treatment (white circle) were challenged with increasing concentrations of Aβ oligomers tagged with HyLite Fluor 488. The percent of synaptosomes with bound Aβ oligomers was determined by flow cytometry analysis. (**c**) Scatchard plot analysis of Aβ oligomer binding show decreased B_max_ values but not Kd values after NIR light treatment in (**c**) cortical synaptosomes and (**d**) hippocampal synaptosomes from WT mice (n = 9 per treatment group; 3 independent pooled samples of 3 mice per group). Statistical significance was determined by Student’s two tailed t-test analysis on three separate binding curve analysis of three to four pooled samples per group. *p < 0.05.

To investigate the effects of NIR light on synaptic sensitivity to Aβ, we studied the *ex vivo* Aβ oligomer binding on isolated synaptosomes from wild type mice receiving NIR light treatment. The NIR light treatment consisted of a 90 second exposure (Fig. 1) administered once a day, 5 days a week for 4 weeks as detailed in Methods. Using flow cytometry analysis, we performed a binding curve with various concentrations of Aβ oligomers labeled with Flour 488 (from 1 μM to 15 μM). As shown in Fig. 2, we gated for synaptosomes based on size, using appropriate standards (Spherotech, Inc.) (Fig. 2). The parameters were set to include particle sizes that are typical of synaptosomes which range from 1–5 μm, as previously described by Gylys *et al*.^25^. This size parameter insures that the particles included in the analysis are synaptosomal elements. This method allows us the ability to exclude any nonspecific binding of Aβ oligomers onto non-synaptosomal particles that may be present in the synaptosomal prep. We found that the pooled (3 pooled samples per group, consisting of 3 individual mice per sample for a total of 9 mice per group) hippocampal and cortical synaptosomal fractions from mice treated with NIR light had decreased Aβ binding at the concentrations of Aβ oligomers used (Fig. 3a,b). We further confirmed this reduced binding affinity using the an Aβ_1–42_ specific ELISA analysis (Supplementary Fig. 1). These results strongly suggest that NIR light treatment reduces synaptic susceptibility to Aβ oligomer binding. By further analyzing the data using Scatchard plot analysis (Fig. 3c,d), we found that the maximum binding capacity (B_max_) was reduced in the group receiving NIR light treatment as compared to the sham-treated group in both the hippocampus (NIR-treated B_max_ = 79.99 +/− 3.18 μM and sham treated group B_max_ = 98.07 +/− 9.84 μM, p < 0.05) and the cortex (NIR-treated group B_max_ = 87.58 +/− 8.40 μM and sham treated group B_max_ = 63.14 +/− 17.46 μM, p < 0.05). On the other hand, no changes in the affinity (Kd) of Aβ binding were observed between groups for either the cortex or hippocampus (Kd = 5.7 +/− 1.95 μM and 9.00 +/− 5.08 μM, respectively, for the NIR-treated group and Kd = 5.12 +/− 1.97 and 8.14 +/− 4.22 μM, respectively, for the sham-treated group). The smaller B_max_ along with unchanged Kd indicates that there was a reduction in the number of binding sites at the synapses in the group that received NIR light treatment.

**Figure 4:**
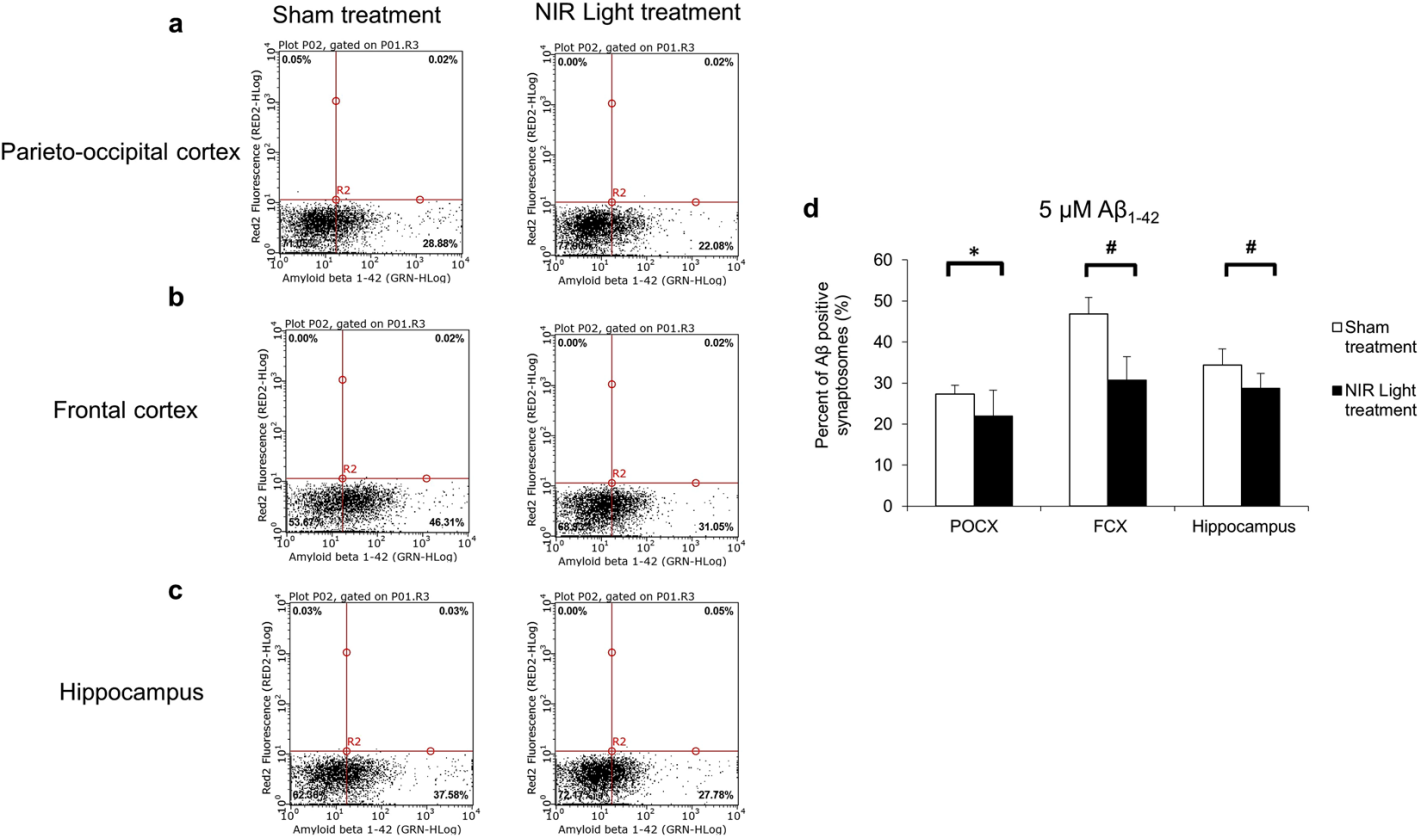
Flow cytometry analysis of *ex vivo* synaptic binding of 5 μM Aβ oligomers. Representative flow cytometry analysis of the 5 μM Aβ oligomer binding in synaptosomes isolated from (**a**) parieto-occipital cortex (POCX) (**b**) frontal cortex (FCX) and (**c**) hippocampus in NIR light treated and sham treated wild type mice. (**d**) The results indicate a significant reduction in the percentage of Aβ positive synaptosomes in the three brain regions POCX, FCX and hippocampus of NIR light treated mice (black) compared to sham treated mice (white). (n = 8; per group). Statistical significance was determined by Student’s two tailed t-test analysis. Error bars represent standard deviation. *p < 0.05; ^#^p < 0.01.

We also performed the *ex vivo* Aβ oligomer binding at a single concentration (5 μM) on synaptosomes prepared from each individual wt mouse (n = 8; per group) to further demonstrate changes in binding properties due to NIR light treatment. Consistent with the results described above, we found that in the parieto-occiptial cortex, frontal cortex, and hippocampus there was a significant reduction of Aβ_1–42_ positive synaptosomes of about 7%, 16%, and 6%, respectively, in the samples prepared from NIR light treated mice as compared to the control, sham-treated animals (Fig. 4a–d).

### Decreased Aβ oligomer level in total homogenate and at the synapses in 6 month old Tg2576 mice after treatment with NIR light

**Figure 5:**
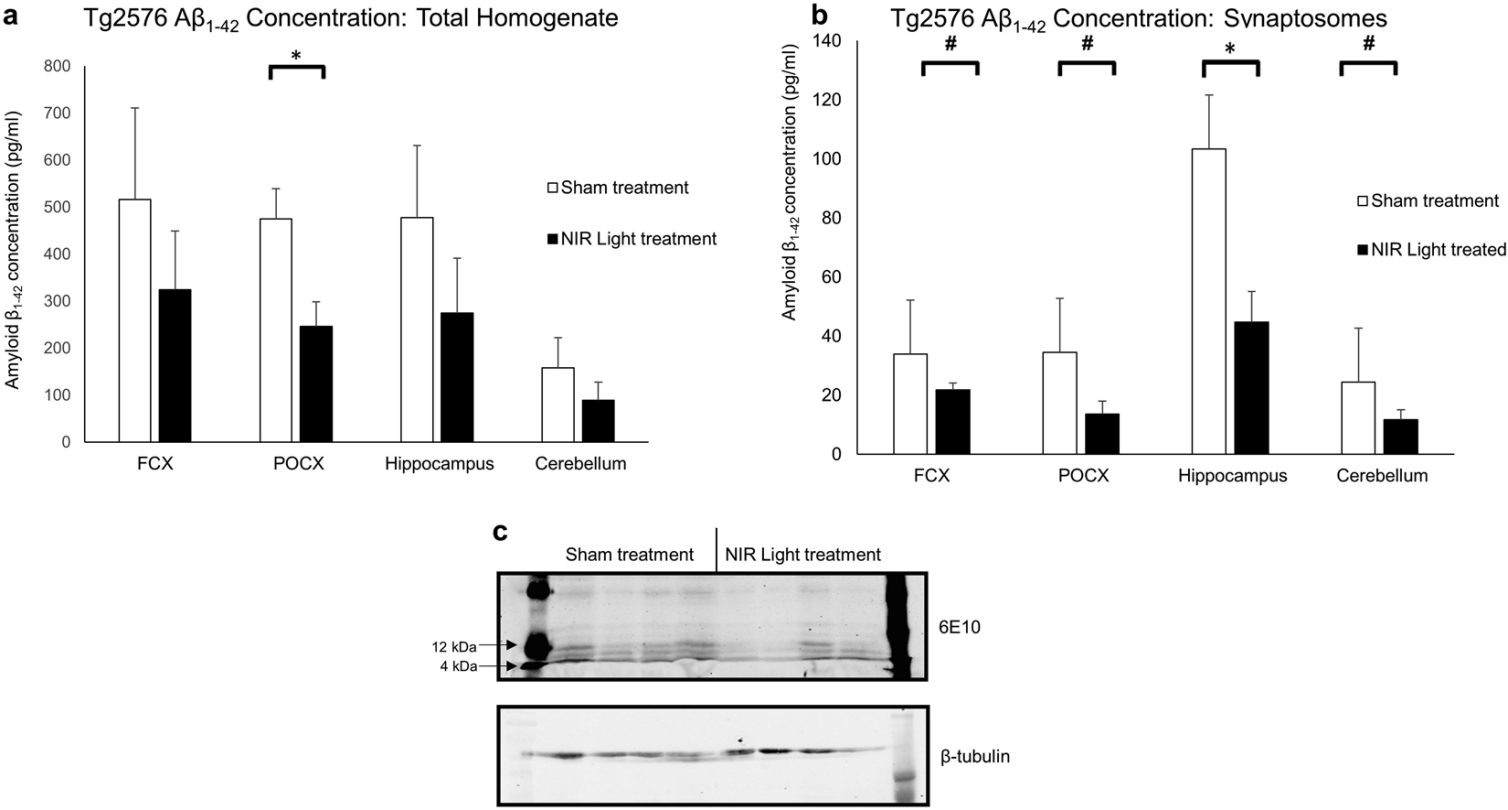
NIR light treatment decreases Aβ_1–42_ oligomer levels in total homogenate and at the synapses in 6-month-old Tg2576 mice. Aβ levels in (**a**) total homogenate and (**b**) synaptosomes from four major brain regions; frontal cortex (FCX) parieto-occipital cortex (POCX), hippocampus and cerebellum of NIR light (filled columns) and sham (open columns) treated Tg2576 mice. (n = 7 per group) were measured using ELISA analysis. (**a**) NIR light treated mice had decreased levels of Aβ_1–42_ the total homogenate from the POCX compared to wt mice. (**b**) NIR light treated mice also had decreased levels of Aβ_1–42_ in the synaptosomes isolated from all of the four brain regions assayed (POCX, FCX, hippocampus and cerebellum). (**c**) Western blot analysis was further used to determine the changes in the low molecular weight Aβ oligomers after NIR light treatment. Student’s two tailed t-test was used to determine statistical significance. Error bars represent SEM. *p < 0.05; ^**#**^p < 0.01.

In order to determine the effect of NIR light treatment in protecting synapses from endogenous Aβ oligomers *in vivo*, we investigated changes in Aβ oligomer levels in synaptosomes and total protein extracts from a well characterized mouse model of AD-like pathology, the Tg2576 mice. In the Tg2576 mouse model, oligomers begin accumulating at the age of 3–5 months and plaques are not observed until the mice are older, around 11–12 months of age^21–23^. NIR light treatment was started at the age of 6 months (n = 7; per group) and the mice sacrificed one month later. Levels of Aβ in the synaptosomal fractions and total homogenates from the frontal cortex, hippocampus, parieto-occipital cortex, and cerebellum were analyzed by a specific ELISA. As shown in Fig. 5b, there was a significant decrease in Aβ levels in the synaptosomal fractions from the four brain regions (parieto-occipital cortex, frontal cortex, hippocampus and cerebellum) in NIR light-treated mice as compared to sham animals. Western blot analysis of the synaptosomes further confirmed changes in levels of the low molecular weight Aβ oligomers (Fig. 5c). On the other hand, a significant decrease of Aβ levels in the total protein extracts was observed only in the parieto-occipital cortex (Fig. 5a) of NIR-treated mice, whereas Aβ levels in the hippocampus, frontal cortex and cerebellum were unchanged, although a non-statistically significant trend toward reduction of Aβ levels in these regions was noticed.

### NIR light reduces Aβ oligomer-induced deficits in long term potentiation

**Figure 6:**
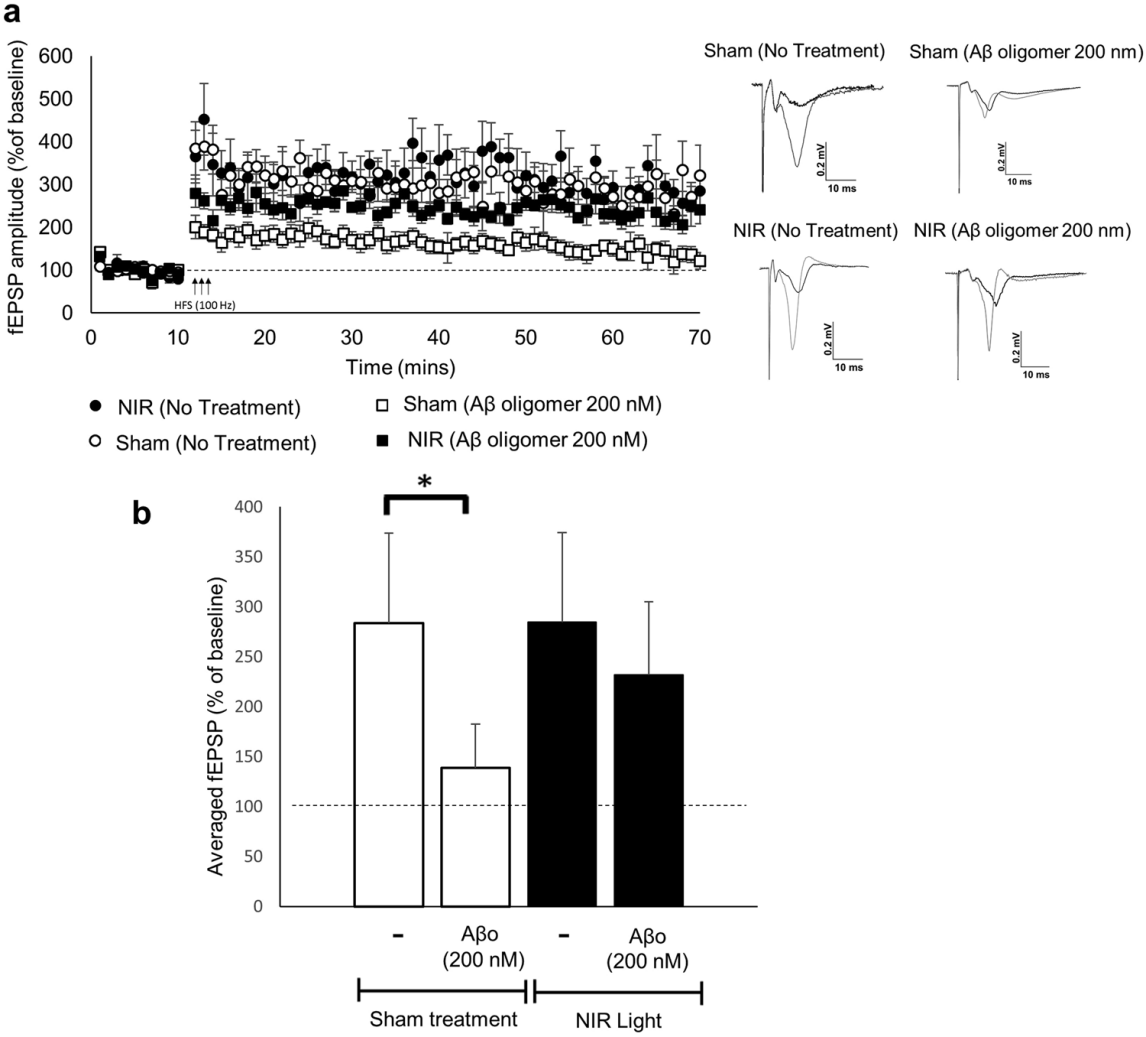
Aβ oligomer driven LTP impairment is reduced with NIR light treatment. Schaffer collateral field recordings were performed to determine NIR light treatments impact on LTP in the presence of Aβ oligomers. (**a**) The percent of baseline in the slope of fEPSPs of NIR light treated wild type mice compared to sham treated wild type mice exposed to Aβ oligomers (n = 5; per group, 2 slices per condition). (**b**) The fEPSP amplitude for the final 10 minutes (time points 50–60 minutes post high frequency stimulation) were averaged for each condition. The sham light treated groups receiving Aβ oligomers had a significant reduction in LTP. This reduction was reversed in the group that received NIR light treatment. One way ANOVA with Dunn’s post hoc analysis was used to determine statistical significance. Error bars represent ± standard error of mean. *p < 0.05.

In order to determine whether the reduced susceptibility of synapses to Aβ oligomers would translate into functional protection, we utilized wild type mice to determine if NIR light treatment rescues the synaptic impairment of LTP that normally occurs in response to exposure to Aβ oligomers^26^. As shown in Fig. 6a, we found that the magnitude of LTP in hippocampal slices from sham-treated mice was significantly reduced by exposure to 200 nM preformed Aβ oligomers. On the other hand, the magnitude of LTP in hippocampal slices from NIR light-treated animals was not affected by Aβ oligomers and remained comparable to the LTP observed in slices from both the NIR light-treated and sham animals that did not receive Aβ oligomers. In any case, the basal synaptic strength was not altered by NIR light treatment or exposure to the Aβ oligomers across our experimental groups, as determined by input-output curves (Supplementary Fig. 2). When the last 10 minutes of LTP were averaged for each group and statistically analyzed (Fig. 6b), we confirmed a significant reduction of LTP induced by Aβ oligomers in hippocampal slices from sham-treated animals but not in slices from NIR light-treated mice that maintained a level of LTP comparable to control slices.

### NIR light treatment increases synaptic mitochondrial membrane potential in both wild type and Tg2576 mice

**Figure 7:**
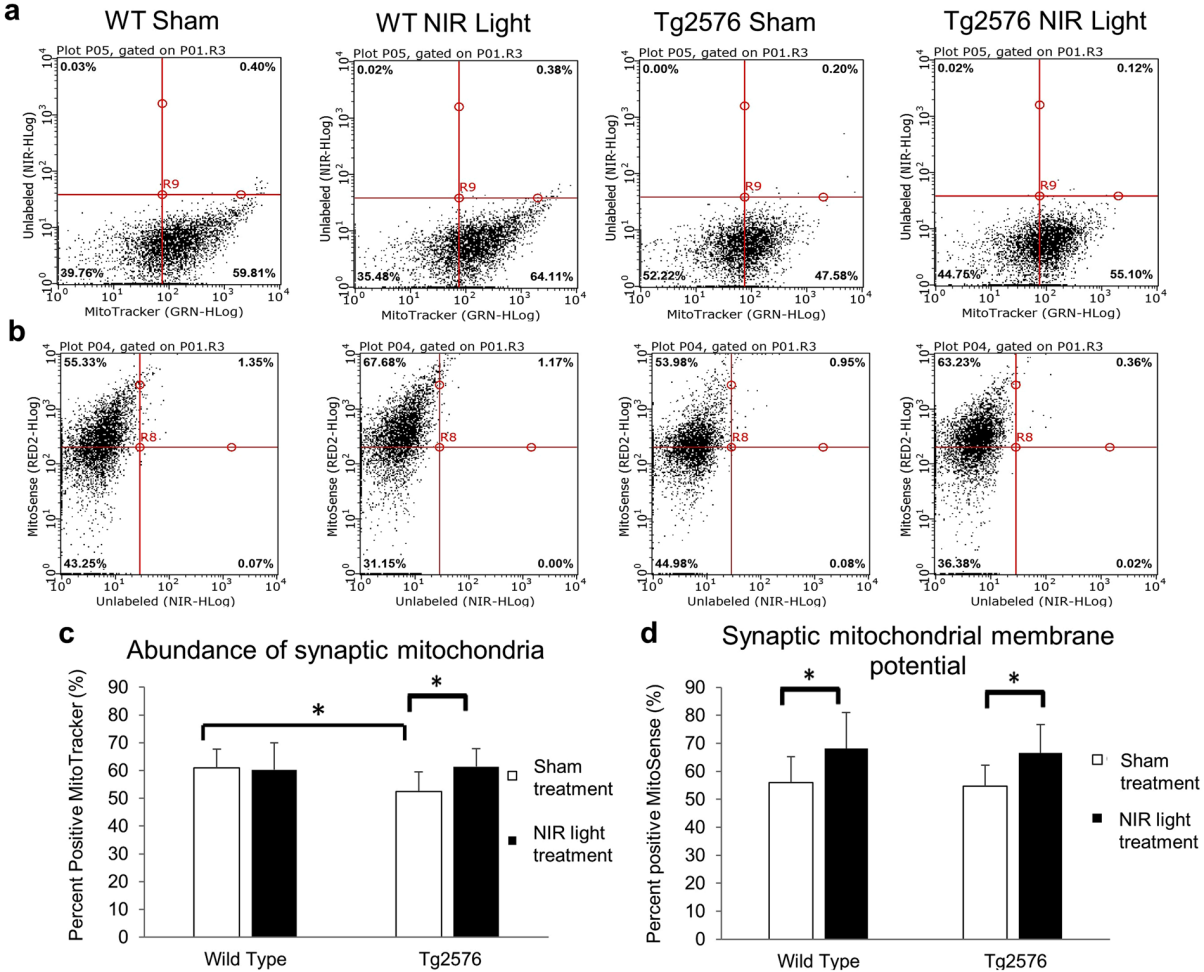
NIR light treatment increases synaptic mitochondrial membrane potential in both wild type and Tg2576 mice and rescues number of synaptic mitochondria in Tg2576 mice. Representative flow cytometry analysis of (**a**) MitoTracker (mitochondria number) positive synaptosomes and (**b**) MitoSense (mitochondria membrane potential) positive synaptosomes of the four groups including wild type and Tg2576 mice treated with NIR light or sham light treatments (n = 8; per group). (**c**) The percentage of positive synaptosomes for MitoTracker is significantly reduced in Tg2576 sham treated mice (white). The Tg2576 mice receiving NIR light treatment (black) have an increase in synaptic mitochondria number resulting in comparable mitochondria numbers to wild type mice. (**d**) The percentage of positive synaptosomes for MitoSense is increased in both wild type and Tg2576 mice treated with NIR light. One-way ANOVA followed by Tukey’s post hoc test was used to determine statistical significance. Error bars represent standard deviation. *p < 0.05.

In these experiments, we focused on synaptic mitochondria function as one possible mechanism contributing to the observed synaptic resilience to Aβ oligomers induced by NIR light exposure. Previous studies have described the mitochondria as a key element targeted by NIR light treatment^10,11,27,28^ and reported that preserving synaptic mitochondria efficiency protects synapses from Aβ oligomers^20,28^. We first analyzed by flow cytometry synaptosomes from wild type and Tg2576 mice treated or not with NIR light that had been labeled with MitoTracker, a mitochondrion-specific fluorescent dye whose stain intensity is directly proportional to the number of mitochondria^29^. We found that there was a decreased number of synaptic mitochondria in Tg2576 mice as compared to age matched wild type mice (Fig. 7a,c). However, Tg2576 mice receiving NIR light treatment had an abundance of mitochondria at the synaptosomes comparable to wild type mice. We further investigated the mitochiondrial membrane potential (reflecting overall mitochondrion health) of the synaptic mitochondria by labeling synaptosomes with the fluorescent dye MitoSense, which directly correlates with the levels of mitochondria membrane potential^30^. Using flow cytometry in labeled synaptosomes we observed an increase in the mitochondrial membrane potential in both the wild type and Tg2576 mice treated with NIR light as compared to sham-treated controls (Fig. 7b,d), suggesting that the NIR light treatment increases overall health of synaptic mitochondria in both wt and Tg2576 mice.

## Discussion

Synaptic function is an essential element in the maintenance and preservation of cognition^31–35^. Aβ oligomer preferential binding to the synaptic region disrupts synaptic transmission and contributes to the declining functionality of the synapses during the progression of AD^36,37^. The main goal of the current study was to determine the ability of NIR light to mitigate the toxic binding of Aβ oligomers to the synapses, thus alleviating the resulting synaptic dysfunction. Our *ex vivo* Aβ oligomer challenge model allowed us to exclusively isolate the synaptosomes after NIR light treatment and determine differences in Aβ binding between the groups. By directly adding prepared Aβ oligomers to isolated synaptosomes, we could demonstrate acquired synaptic resistance to Aβ oligomers binding after NIR light exposure. We found that wt mice treated with NIR light have a reduction in the amount of Aβ oligomers that bind to the synaptosomes. The results of our binding curve provided us the opportunity to characterize the binding changes that were occurring after NIR light treatment. We used Scatchard plot analysis to calculate the Aβ oligomer maximum binding capacity and affinity in the two experimental groups (NIR-treated and sham-treated). The decreased B_max_ value in the NIR light treated groups with no change in the affinity (Kd) suggests that after NIR light treatment there is a reduction in Aβ oligomer docking sites at the synapse, providing compelling evidence that a secondary mechanism of action of the NIR light treatment is contributing to the increased resilience to the toxic protein. By gating our observation to selected particle sizes, flow cytometry further allowed us to exclude nonspecific binding to nonsynaptosomal particles.

We utilized the Tg2576 mouse strain that overexpresses human APP and progressively accumulate Aβ oligomers^21–24^ to determine whether an *in vivo* reduction of endogenous Aβ at the synapses could be observed after NIR light treatment. Previous research has shown a reduction of the Aβ plaque load after NIR light treatment in the cortex and hippocampus of APP/preselinin 1 (PS1) double transgenic mice^38^. However, this previous study focused on overall Aβ levels in plaques and did not address the effect of NIR light on synapse vulnerability to the toxic Aβ oligomers, as we have determined here. To provide a complete understanding of the impact NIR light has on synaptic vulnerability to Aβ, in our study Tg2576 mice were treated with NIR light and the Aβ levels were measured in both total brain protein extracts as well as synaptosomal fractions. We employed Tg2576 mice at age of 6 months when ample Aβ load, mainly in the form of oligomers, is seen and cognitive defects are prevalent, but plaques formed by large Aβ fibrils deposition are not yet present^22–24^. Therefore, Tg2576 mice at 6 months of age are an ideal model to study specifically the impact of Aβ oligomers. Our results showed a significant decrease of Aβ load in the synaptosomes from all four brain areas tested, i.e. parieto-occipital cortex, hippocampus, frontal cortex and cerebellum. However, the total brain tissue homogenates showed a significant decrease of Aβ only in the parieto-occipital cortex. The other three brain regions showed a trend of decreased levels, however, they reduction was not statistically significant. The differential impact of the NIR light among the brain regions may be attributed to higher energy exposure to superior structures or higher sensitivity to NIR light of the parietal-occipital cortex, although further experiments are needed to support either (or both) of these possibilities. The differences in reduction between the total homogenate and synaptosomes suggest that NIR light initiates a mechanism that targets specifically the synapses. Interestingly, this selective reduction of Aβ at synapse induced by the NIR light treatment is similar to what observed in the cognitively intact NDAN individuals. As we have previously described, NDAN individuals have presence of Aβ oligomers in the CNS at levels similar to demented AD patients, however, they have an absence of Aβ oligomers at the synapses, which suggests synaptic resistance to oligomer binding^7^. The results of our *in vivo* experiment thus suggest that NIR light treatments may effectively evoke a mechanism of synaptic resilience similar to the natural ability to resist the binding of Aβ oligomers seen in NDAN individuals with preserved cognitive integrity.

We further aimed to relate this molecular phenomenon of NIR-induced resilience with synaptic functional outcomes as an indication of preserved synaptic efficiency. The underlying deficiency of cognition in AD is the loss of synaptic function^1,34,35,39^. A well-established impairment that occurs when Aβ oligomers bind to synapses is reduced magnitude of LTP^40^ and increased long term depression (LTD)^41^. In the present study, we aimed to determine if NIR light reversed this observed reduction of LTP expression by Aβ oligomers. Consistent with those previous experiments, we found a significant reduction in the magnitude of LTP in wt mice incubated with 200 nM of Aβ oligomers. However, after NIR light treatment wt mice demonstrated an induction of LTP similar to the control group without Aβ oligomer exposure. This suggests that the reduction of Aβ oligomer binding that we observed following NIR light treatment in the *ex vivo* studies results in a significant rescue in synapse functionality. The electrophysiology experiment was conducted after treating the wt mice with a condensed treatment protocol. We found that mice receiving the same number of treatments (4 treatments per day for 5 days; 20 total doses of NIR light) and the same delivery of total energy as the month-long treatment over a course of 5 days showed similar reductions in the synaptic binding of Aβ oligomers (Supplementary Fig. 3). While there are a few studies that have investigated the total energy from NIR light needed to have a biological impact on the system^42^, little is known about the persistence of NIR light’s beneficial effects after the end of a complete treatment cycle. Future studies are needed to address this important issue with significant implications for the translational value of NIR treatment.

A possible mechanism that could be contributing to the protection and increased function of synapses seen in our study is increased function of the synaptic mitochondria. The 600–1000 nm wavelength spectrum of the NIR light has been shown to optimally photostimulate cytochrome c oxidase, a key enzyme in the electron transport chain that is believed to result in an increase in ATP production^9,43,44^. Cytochrome c oxidase contains 2 copper centers and 2 heme iron centers. When photostimulated, the copper centers are unable to bind nitric oxide, an inhibitor of respiration, resulting in an increase of oxygen consumption and ATP formation^9,10,44^. Several previous studies have investigated the dysfunction of mitochondria in AD^44–46^. Notably, there is an impairment in the mitochondrial transport mechanisms^47^ and in the fission/fusion process of mitochondria^48,49^ specifically at the synapses^50^. Collectively, these impairments lead to a decrease in functional synaptic mitochondria resulting in reduced ATP supply, ultimately leaving the synapses vulnerable to toxins such as Aβ oligomers. The reported decrease in mitochondria functionality in AD may thus benefit from the mitochondria-boosting properties of NIR light. In our study, we investigated the effects of NIR light on synaptic mitochondrial membrane potential (reflecting overall mitochondrial health) and synaptic mitochondrial abundance. Consistent with the literature, we found a decrease in the abundance of synaptic mitochondria in Tg2576 mice as compared to age matched wild type mice^48^ and further observed that after NIR light treatment, this deficiency in the synaptic mitochondria was rescued. On the other hand, wild type mice treated with NIR light did not have an increase in synaptic mitochondria compared to their control treatment counterparts. This suggests that under normal physiological conditions, NIR light does not induce an increase of mitochondria numbers at the synapses. However, the rescued synaptic mitochondria number that is seen in the NIR light-treated Tg2576 synapses suggests that the NIR light-induced reduction of Aβ oligomer binding contributes to the reversal of the depletion of synaptic mitochondria. Nonetheless, given the fact that the beneficial effect of NIR light in promoting synaptic resilience to Aβ oligomers was observed in both wt and Tg2576 mice, synaptic mitochondrial abundance does not appear to be a key event in this action of NIR light. On the other hand, when we examined the membrane potential of the synaptic mitochondria as an indicator of mitochondrial health after NIR light treatment, we found that both wild type and Tg2576 mice showed an increase in synaptic mitochondrial membrane potential after treatment with NIR light. Previous studies have shown a direct relationship between synaptic vulnerability to Aβ oligomer binding and the health of synaptic mitochondria^20,28^. Furthermore, synaptic mitochondria are believed to have an integral role in the regulation of LTP induction^51,52^. Therefore, our results suggest a mechanism of NIR light action critically involving increasing synaptic mitochondrial membrane potential, consistent with the reported increase in synaptic resilience to Aβ oligomers induced by promotion of synaptic mitochondrial function/health. Taken together these results indicate that NIR light treatment increases synaptic mitochondria health, thus decreasing synaptic susceptibility to Aβ binding and consequent electrophysiological deficits in LTP expression.

Collectively, the results of our study provide valuable insight into the mechanisms that underscore the reported mitigation by NIR light of AD pathology and further introduce a previously unappreciated phenomenon of increased synaptic resilience to the disrupting action of Aβ oligomers that can be promoted by NIR light exposure.

## Methods

### Animals

Male and female transgenic 2576 (Tg2576) mice were utilized to evaluate *in vivo* Aβ load at the synapses (n = 7, per experimental group), as well, as the electrophysiological properties after NIR light treatments (n = 5, per experimental group, 2 slices per animal per experimental condition). C57BL/6 wild-type male and female mice were employed for the *ex vivo* Aβ binding experiments (n = 8, per experimental group). Male and female Tg2576 and wild type mice were utilized for synaptic mitochondrial experiments (n = 8, per group). All experimental protocols involving animals were approved by Institutional Animal Care and Use Committee of the University of Texas Medical Branch. All methods were performed in accordance with the guidelines and regulations of the committee. Animals were housed under USDA standards (12:12 hour light dark cycle, food and water *ad libitum*) at the UTMB vivarium.

After the conclusion of the NIR light treatment, the mice were sacrificed by exposure to isoflurane and decapitated. The brains were quickly removed, dissected into major regions; frontal cortex, parieto-occipital cortex, hippocampus and cerebellum and stored at −80 °C until ready for further analysis.

### NIR light treatments

One dose of the NIR light treatment consisted of a 90 second treatment from a 670 nm light-emitting diode (LED) device (WARP 10; Quantum Devices, Barneveld, WI, USA). Light energy emitted equated to 4 Joule/cm^2^ per treatment. The treatment group received one dose per day, 5 days per week for 4 consecutive weeks. The mice were hand restrained and the light device was held approximately 1 cm from the top of the head. The body of the mouse was covered with aluminum foil to prevent light exposure to the periphery, as illustrated in Fig. 1. The control sham light treated group was restrained in the same manner as the treatment group, however the light device remained off, as previous described^38^. The NIR light treatment was condensed to one week (4 treatments per day for 5 days) for wt mice treated for the electrophysiology experiment after it was determined that this condensed time period provided similar reduced binding effects as the 4 week treatment schedule (Supplementary Fig. 3).

### Isolation of Synaptosomes

After the final NIR light treatment, the animals were sacrificed and the brains collected as described above. To isolate synaptosomes, the tissue was homogenized in Syn-PER synaptic protein extraction reagent (ThermoFisher). A portion of the total homogenate was saved for biochemical analysis and the remaining portion was centrifuged at 1200 × g for 10 minutes at 4 °C. The supernatant was collected and centrifuged further at 15000 × g for 20 minutes at 4 °C, as per reagent instructions. The synaptosomes containing pellet were resuspended in HEPES-buffered Krebs-like (HBK) buffer or radioimmunoprecipitation assay (RIPA) buffer depending on future experiment. The quality of the synaptosomal isolation is routinely verified by Western blot and electron microscopy, as shown by Franklin, 2016^53^.

### Ex vivo amyloid β oligomer binding

#### Aβ oligomer preparation

Human Aβ oligomers were prepared from lyophilized synthetic Aβ aliquots (0.3 mg) dissolved in 0.2 ml of 1,1,1,3,3,3- Hexafluro-2-propanol (HFP). The HFP- Aβ mixture was then added to 0.7 ml of H_2_O. A cap with four holes was placed on the tube and the sample was stirred by a magnetic stir bar under a fume hood for 48 hours. The sample was used immediately after the 48 hours of stirring^54^. For the flow cytometry analysis of Aβ oligomer binding to the synaptosomes, Aβ oligomers with a Flour 488 tag were utilized. These Aβ oligomers were prepared by adding Aβ_1–42_ peptide with a Flour 488 tag (AnaSpec, Inc) to the HFP-Aβ mixture described above. This mixture was then added to 0.7 ml of H_2_O and spun, as described. Western blot and dot blot analysis using A-11 antibodies (Aβ oligomer specific) are used to determine the quality of oligomerization (previously described by Reese (2008)^55^.

#### Aβ oligomer binding challenge

The *ex vivo* binding of Aβ oligomers to the synaptosomes of mice treated or not with NIR light was evaluated using flow cytometry. Synaptosomal fractions of male and female wild type mice receiving NIR light treatment were prepared and resuspended in HBK buffer. Synaptosomal fractions from multiple animals in each group were combined in separate tubes. Pooled samples were then aliquoted into 8 separate tubes per group containing 100 mg of total protein per tube determined by bicinchoninic acid (BCA) assay. To perform analysis using flow cytometry, the synaptosomes were prepared and aliquoted as described in the ELISA analysis, however, the synaptosomes were treated for 60 minutes with Aβ oligomers tagged with HyLite Fluor 488 (AnaSpec) in various concentrations ranging from 1 μM to 10 μM. The synaptosomes were then centrifuged and washed multiple times with HBK buffer and resuspended in PBS. Data was acquired by a Guava EasyCyte flow cytometer (EMD Millipore) and analyzed using Incyte software (EMD Millipore).

### Measurement of *in vivo* amyloid β load

Synaptosomes and total homogenate were prepared from Tg2576 mice as described above and resuspended in RIPA buffer. Western blot analysis was performed on these protein extracts to determine the presence of Aβ oligomers. Separation of the proteins in the samples obtained was done by 12% SDS- polyacrylamide gel electrophoresis. The separated proteins were transferred to a nitrocellulose membrane (Bio-Rad) and incubated with 1:1000 6E10 (total Aβ) antibody overnight. The nitrocellulose membrane was then incubated with the appropriate fluorescent secondary antibody and imaged with an Odyssey infrared imager. The band densities were analyzed using Image J software, normalizing using the densities of the loading control obtained by reprobing the membranes for β-tubulin. Quantification of Aβ levels in the protein extracts was determined using an Aβ_1–42_ specific solid phase sandwich enzyme-linked immunosorbent assay kit (ELISA) (Invitrogen).

### Electrophysiology

The wt mice were anesthetized with isoflurane and were perfused intracardially with a NMDG solution containing; 93 mM NMDG, 2.5 mM KCl, 1.2 mM NaH_2_PO_4_, 30 mM NaHCO_3_, 20 mM HEPES, 25 mM glucose, 5 M sodium ascorbate, 2 mM thiourea, 3 mM sodium pyruvate, 10 mM MagSO_4_ ·7H_2_0, 0.5 mM CaCl_2_ ·2H_2_O, and 12 mM N-acetyl L-Cysteine. Brains were harvested and sliced in the NMDG solution followed by a 10-minute recovery period in 35 °C NMDG solution. The slices were then maintained in a modified HEPES holding aCSF (92 mM NaCl, 2.5 mM KCl, 1.2 NaH_2_PO_4_, 30 mM NaHCO_3_, 20 mM HEPES, 25 mM Glucose, 5 mM sodium ascorbate, 2 mM thiourea, 3 mM sodium pyruvate, 2 mM MgSO_4_ 7H_2_0, 2 CaCl_2_ 2H_2_0, 12 N-Acetyl L-Cysteine). For recording the slices where held in an artificial cerebrospinal fluid (aCSF) (124 mM NaCl, 2.5 mM KCl, 1.2 mM NaH_2_PO_4_, 24 mM NaHCO_3_, 5 mM HEPES, 12.5 mM glucose, 2 mM MgSO_4_·7H_2_0 and 2 mM CaCl_2_·2H_2_0) (Ting, 2014). All solutions were supplemented with 95% O_2_/5% CO_2_. Prior to recording, the slices were incubated with 200 nM of Aβ oligomers for 1 hour at room temperature. Field excitatory post-synaptic potentials (fEPSPs) recordings were performed by stimulating the Schaffer collateral pathway. The stimulating electrode of 22 kΩ resistance was placed in the cornu ammonis 3 (CA3) region and the recording electrode was located at the junction of the alveus and cornu ammonius 1 (CA1). High frequency stimulation consisting of 3–100 Hz trains for 1 second duration with 20 second intertrain intervals was be used to induce LTP. The traces were analyzed with Clampfit 10.6 software (Molecular Devices).

### Measurement synaptic mitochondrial membrane potential and abundance of synaptic mitochondria

The synaptosomes were isolated from frozen brain tissue as described earlier. The synaptosomes were then immediately treated with Mitotracker green FM (Invitrogen) and MitoSense Red (1,1′,3,3,3′,3′- Hexamethylindodicarbocyanine iodide) (EMD Millipore) for 15 minutes at 37 °C. MitoTracker is a fluorescent dye that diffuses across the mitochondrial membrane and reacts with thiol groups of specific mitochondrial proteins^29^. The fluorescent dye MitoSense correlates with mitochondria membrane potential^30^. The synaptosomes were washed twice with HBK buffer and fluorescent emittance was acquired by a Guava EasyCyte flow cytometer (EMD Millipore) and analyzed using Incyte software (EMD Millipore).

### Statistical Analysis

The statistical analysis of the data was analyzed using SPSS software. Student’s t test was used to determine statistical significance.

## Electronic supplementary material

Supplementary Information
