# Supplementary material for: Near infrared light decreases synaptic vulnerability to amyloid beta oligomers
Source: Sci Rep. 2017 Nov 8;7:15012. doi: 10.1038/s41598-017-15357-x (PMC5678170; doi:10.1038/s41598-017-15357-x)
Supplement: Supplementary file 1 — Supplementary Information [file 41598_2017_15357_MOESM1_ESM.pdf]

Supplementary Figures

for

Near infrared light decreases synaptic vulnerability to amyloid beta oligomers

Michele M. Comerota, Balaji Krishnan, Giulio Taglialatela

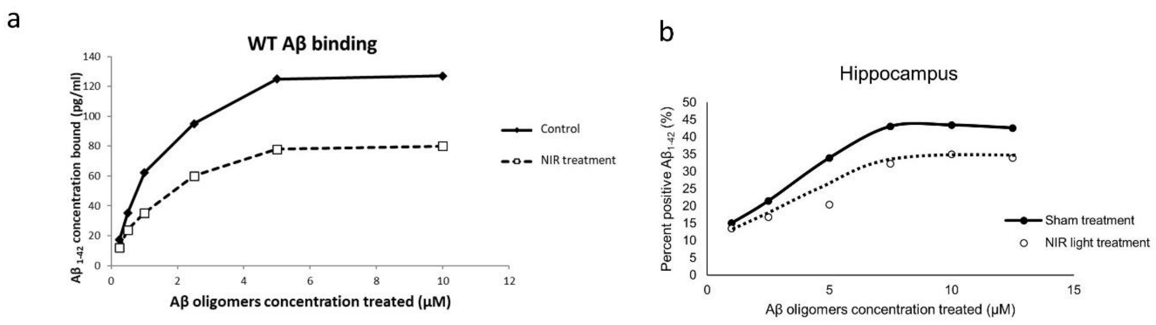

Supplementary Figure 1. ELISA and flow cytometry Aβ oligomer binding curves

Two methods were imploded in the determination of changes in the Aβ oligomer binding between NIR light treated and sham control treated wild type mice; ELISA and flow cytometry analysis. The flow cytometry analysis as described in the Methods section was used in determining the percentage of synaptosomes in our synaptosomal prep that would bind a fluorescently tagged Aβ oligomer. The ELISA method was similar, however, the Aβ oligomers were prepared without a fluorescent tag, so the analysis determined the total amount of Aβ oligomers bound in our sample. The flow cytometry method was chosen as the main focus in our current study, because of the added ability of the method to selectively analyze the synaptosomes in our prep, excluding nonspecific binding of the tagged Aβ oligomers to nonsynaptosomal particles. As shown in this figure, both methods illustrated a reduction of binding in the NIR light treated mice compared to the control sham group. Further both methods demonstrated a saturation of Aβ oligomer binding to isolated synaptosomes, thus further confirming overall validity of the ex vivo binding procedure used here.

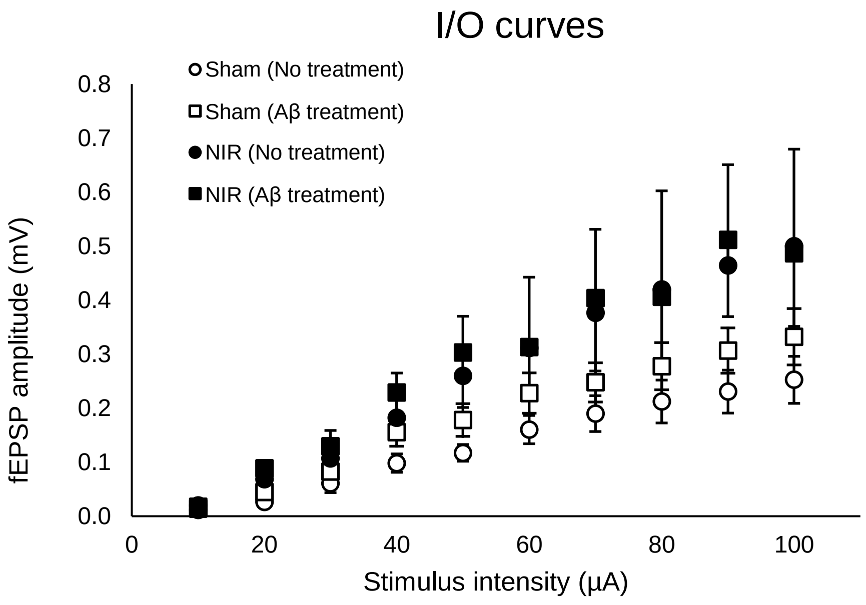

Supplementary Figure 2. Input/output curves for the four treatment groups.

The fEPSP amplitude (mV) obtained at increasing stimulus intensities (mA) show no significant differences in the basal synaptic strength following NIR light treatment and/or exposure to Aβ oligomers compared to sham (no treatment). n=6-8 slices from 3-6 mice; Statistical analysis was carried out using two-way ANOVA with Bonferroni post-hoc analysis, $F_{9,3}$=0.4209, P=0.9954, ns. Error bars represent standard error of mean.

MEAN (n=6)	Sham (No treatment)		Sham (Aβ treatment)		NIR (No treatment)		NIR (Aβ treatment)	
mV	Pre	Post	Pre	Post	Pre	Post	Pre	Post
10	0.012	0.034	0.018	0.060	0.020	0.056	0.014	0.081
20	0.026	0.033	0.045	0.046	0.069	0.034	0.089	0.082
30	0.061	0.077	0.083	0.083	0.107	0.140	0.129	0.167
40	0.098	0.107	0.156	0.139	0.182	0.175	0.229	0.302
50	0.117	0.140	0.178	0.163	0.260	0.258	0.303	0.308
60	0.160	0.174	0.228	0.189	0.311	0.292	0.313	0.382
70	0.190	0.215	0.248	0.226	0.376	0.360	0.404	0.455
80	0.212	0.248	0.278	0.262	0.420	0.390	0.406	0.483
90	0.231	0.262	0.307	0.273	0.464	0.424	0.512	0.550
100	0.252	0.255	0.332	0.299	0.500	0.431	0.487	0.559

SEM (n=6)	Sham (Untreated)		Sham (Abeta Treated)		NIR (Untreated)		NIR (Abeta Treated)	
mV	Pre	Post	Pre	Post	Pre	Post	Pre	Post
10	0.006	0.009	0.006	0.018	0.006	0.014	0.005	0.030
20	0.006	0.011	0.012	0.014	0.034	0.014	0.031	0.035
30	0.017	0.017	0.018	0.022	0.052	0.041	0.054	0.048
40	0.017	0.026	0.026	0.023	0.083	0.075	0.074	0.074
50	0.015	0.023	0.030	0.026	0.110	0.106	0.102	0.085
60	0.026	0.036	0.037	0.026	0.131	0.112	0.086	0.097
70	0.033	0.047	0.036	0.031	0.155	0.132	0.135	0.136
80	0.040	0.055	0.044	0.028	0.183	0.139	0.125	0.134
90	0.040	0.061	0.042	0.038	0.187	0.151	0.142	0.160
100	0.044	0.058	0.052	0.040	0.180	0.156	0.136	0.155

Supplementary Table 1. Table of input/output averages for the four treatment groups.

The averages of the amplitudes (mV) measured in the four treatment groups after increasing stimulus intensities. There was no change in the pre-HFS and post-HFS amplitudes for all four treatment groups. n=6-8 slices from 3-6 mice; Statistical analysis was carried out using two-way ANOVA with Bonferroni post-hoc analysis (Pre-HFS – $F_{9,3}$=0.34799, P=0.9991, ns; Post-HFS - two-way ANOVA, $F_{9,3}$=0.4209, P=0.9954, ns; Pre- vs Post- $F_{9,7}$=0.3395, P=1, ns).

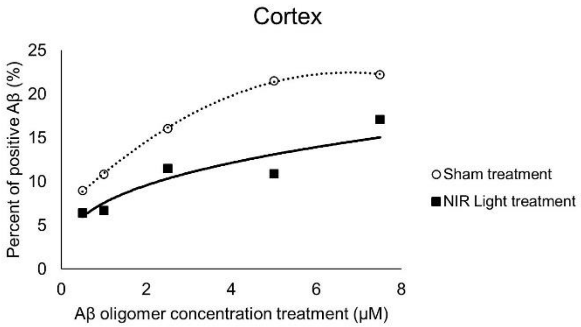

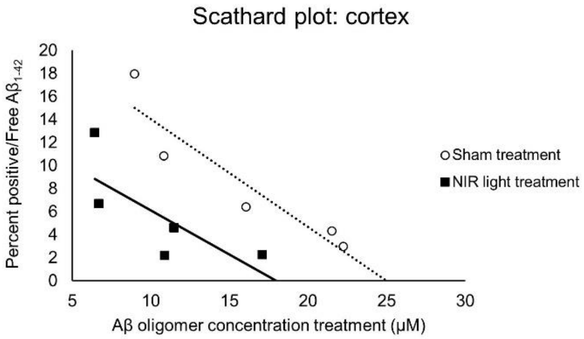

Supplemental Figure 3. Flow cytometry analysis of condensed NIR light treatment regimen Aβ oligomer binding curve.

Pooled synaptosomes from cortex of WT mice receiving a condensed NIR light treatment schedule (20 treatments over 5 days) (black square) had a similar reduction in Aβ binding compared to sham treated mice (white circle) that was demonstrated in WT mice receiving 20 treatments over 4 weeks. Because the synaptosomes of the mice receiving a condensed schedule treatment regimen displayed similar reductions in binding, this schedule was used before performing electrophysiology experiments.

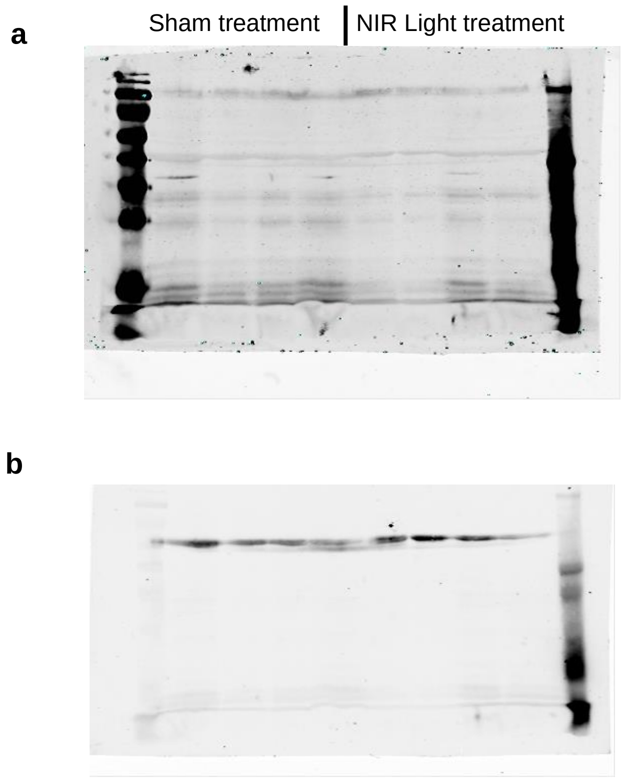

Uncropped Western blot analysis of Tg2576 mice receiving NIR light treatment or sham treatment (Figure 6).

(**a**) Representative Western blot probed with 6E10 antibody. (**b**) The membrane was reprobed using the antibody β-tubulin to serve as a total loading control for each sample.
